# Acitretin-induced psoriasis and Darier disease treated with adalimumab^؆^

**DOI:** 10.1016/j.abd.2025.501267

**Published:** 2026-01-07

**Authors:** Daniel Javier Sánchez-Báez, Júlia Mercader-Salvans, María Luisa Santos e Silva Caldeira-Marques, María del Mar Pestana-Eliche

**Affiliations:** Department of Dermatology, University Hospital of the Canary Islands, Santa Cruz de Tenerife, Spain

Dear Editor,

The simultaneous occurrence of several dermatoses in the same patient supposes a diagnostic-therapeutic challenge for the dermatologist. Although the relationship of psoriasis with other entities is widely known, its association with Darier's disease is a rarity.

We present the case of a 44-year-old woman with a history of sulfonamide allergy, bronchial asthma, and Darier-White disease (Fig. 1) who started treatment with acitretin. The diagnosis of Darier-White disease was confirmed, with histopathology revealing acantholysis with dyskeratosis (numerous grains and corps ronds), as well as parakeratosis (Fig. 2A). After a few months, the patient presented with papules and erythematous-violaceous plaques with silvery desquamation on the extremities. The histopathology showed psoriasiform epidermal hyperplasia, regular acanthosis, parakeratosis, and hypogranulosis, as well as Munro-Sabouraud microabscesses and Kogoj pseudopustules (Fig. 2B), confirming the diagnosis of psoriasis (Fig. 3). Treatment with methotrexate was started, controlling the psoriasis, but recurrent outbreaks of brownish keratotic papules on the trunk made it necessary to maintain the acitretin. Given the insufficient clinical response, treatment was changed to adalimumab, resulting in almost complete clearing of both conditions (Fig. 4). Adalimumab was initiated using the standard induction dosing for psoriasis (80 mg in the first week, followed by 40 mg subcutaneously the following week). The patient showed lesion clearance at 8-weeks of treatment with a maintenance dose of 40 mg subcutaneously once weekly. The treatment remained effective during the subsequent 5-month follow-up period.

**Fig. 1:**
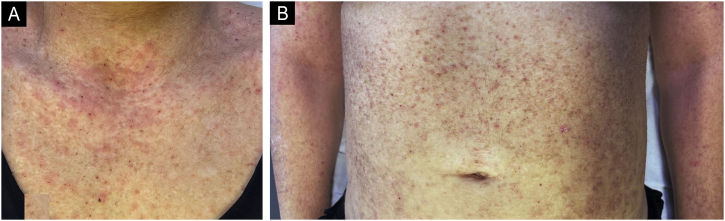
Clinical imaging. Crusted erythematous papules on the neckline (A) and abdomen (B). Clinical findings are compatible with Darier-White disease.

**Fig. 2:**
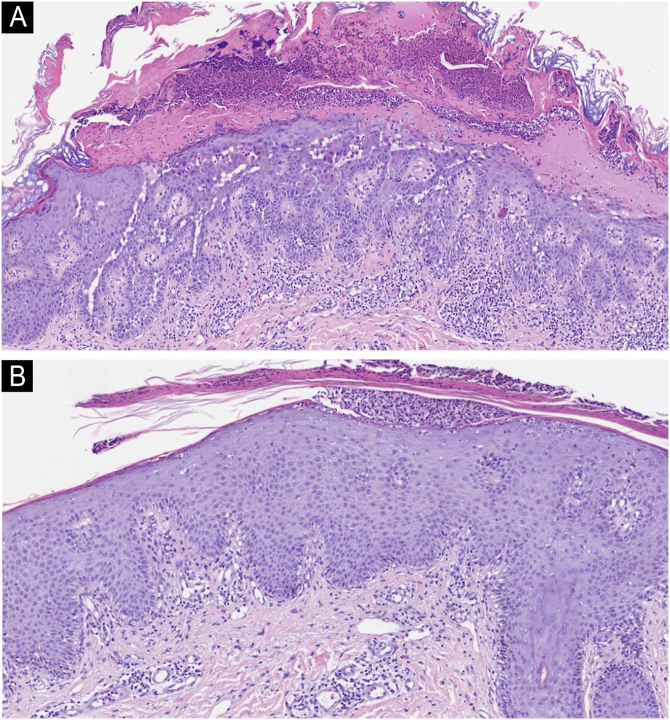
Histological images with hematoxylin and eosin-stained sections (×40). The first image shows acantholysis with dyskeratosis (numerous grains and corp ronds) as well as parakeratosis, findings suggestive of Darier's disease (A). The second image shows regular acanthosis with hypogranulosis and parakeratotic hyperkeratosis, as well as a Munro's microabscess, histologic findings suggestive of psoriasis (B).

**Fig. 3:**
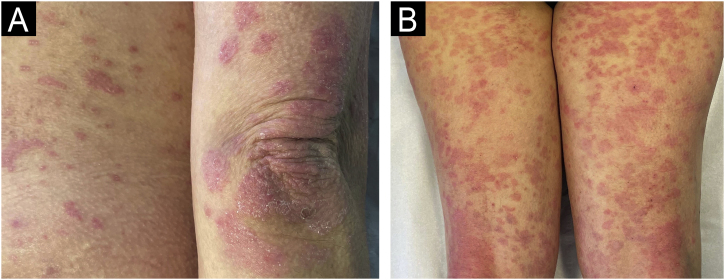
Clinical imaging. Erythematous plaques with silvery-white scaling on elbows (A) and posterior aspect of both lower limbs (B) after initiation of acitretin. Morphologic findings that are compatible with psoriasis.

**Fig. 4:**
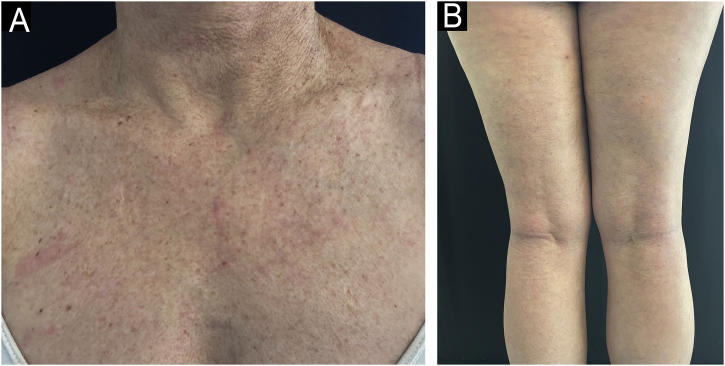
Clinical image. Complete response of both dermatoses after initiation of adalimumab and discontinuation of acitretin at week-8 (A, pre-esternal region; B, legs).

Darier disease (follicular dyskeratosis) is an autosomal dominant genodermatosis associated with a mutation of the ATP2A2 gene, altering intracellular calcium levels.^1^ The estimated prevalence varies from 1:30,000–100,000.^2^ It classically manifests in childhood or adolescence as erythematous-brown and keratotic follicular papules confluent in seborrheic and intertriginous areas.^2^ The disease has a chronic course with exacerbations due to sun exposure, heat, friction, and infections.^2^ Histology shows a characteristic acantholytic dyskeratosis.^2^ Its association with psoriasis is exceptional in the literature, with 2 cases published to date.^3^, ^4^ The debut of psoriasis after initiation of acitretin is also unusual.

The pathophysiologic link between these two diseases has been the subject of several publications. It has been described that patients with psoriasis have a down-regulation of the ATP2A2 gene.^5^ In both diseases, endoplasmic reticulum stress and the consequent response to unfolded proteins are involved in the pathogenesis.^3^ Abnormal expression of involucrin, a protein related to keratinocyte differentiation, was also observed.^6^ Involvement of the Th17-23 axis has been demonstrated in patients with Darier disease.^7^ However, these possible pathogenic associations contrast with the scarce joint report of both conditions.

Secondary psoriasis to acitretin is not documented in the literature. In our case, the Krach and Lasagna algorithm gave us a score of 7, considering probable causality. It has been described in the literature that this retinoid can worsen cases of previous psoriasis, even inducing erythrodermic forms.^8^ Two hundred and seventeen cases of “psoriasis” have been reported with the use of acitretin to the EudraVigilance system of the European Medicines Agency (EMA)^9^ to date. However, the history of the patients and whether the cases were associated with other drugs like biologics is unknown. A possible justification for this reaction could be the hyperproliferative effect that acitretin can have on healthy skin.

Interestingly, the patient was well controlled for follicular dyskeratosis with adalimumab alone. The latter has only been demonstrated for familial benign pemphigus. Only one case of Darier's disease has been published in a patient with rheumatoid arthritis who did not respond to anti-TNF-alpha treatment.^10^

The development of psoriasis in patients with Darier disease is exceptional. Both diseases seem to present common genetic and/or immunologic factors in their pathophysiology, which contrasts with the scarce joint report. Adalimumab could play a role in the treatment.

## ORCID IDs

Júlia Mercader-Salvans: 0000-0001-8662-3003

María Luisa Santos e Silva Caldeira Marques: 0000-0002-5764-6879

María del Mar Pestana Eliche: 0000-0003-2768-6383

## Authors’ contributions

Daniel Javier Sánchez-Báez: Review of the medical literature, image collection, manuscript writing, and image editing with layout design for publication.

Júlia Mercader-Salvans: Supervision and manuscript writing.

María Luisa Santos e Silva Caldeira Marques: Supervision and manuscript writing.

María del Mar Pestana Eliche: Supervision.

## Financial support

None declared.

## Research data availability

Does not apply.

## Conflicts of interest

None declared.
